# Quantitative ¹³C-urea breath test values predict peptic ulcer risk in *Helicobacter pylori* -infected children: a retrospective study

**DOI:** 10.3389/fped.2025.1684120

**Published:** 2025-09-24

**Authors:** Xiaoting Pan, Youtao Chen, Haibo Li, Hong Ye

**Affiliations:** ^1^ Fujian Children’s Hospital, Fujian Branch of Shanghai Children’s Medical Center, College of Clinical Medicine for Obstetrics & Gynecology and Pediatrics, Fujian Medical University, Fuzhou, China; ^2^ Fujian Maternity and Child Health Hospital, College of Clinical Medicine for Obstetrics & Gynecology and Pediatrics, Fujian Medical University, Fuzhou, China

**Keywords:** 13C-urea breath test, *Helicobacter pylori*, pediatric gastroenterology, peptic ulcer, diagnostic accuracy, bacterial load

## Abstract

**Background:**

The quantitative ¹³C-urea breath test (¹³C-UBT) is valuable for diagnosing *Helicobacter pylori* (H. pylori) infection. However, pediatric-specific thresholds and their association with peptic ulcer (PU) disease remain inadequately defined. This study aimed to identify optimal pediatric delta over baseline (DOB) thresholds for diagnosing H. pylori infection and explore associations with ulcer risk in children.

**Methods:**

In this retrospective study, 1,034 consecutive children aged 3–18 years undergoing ¹³C-UBT with endoscopy and histopathological evaluation at Fujian Children's Hospital (May 2021–May 2025) were enrolled. DOB cutoff values were determined by ROC analysis. Logistic regression and restricted cubic spline (RCS) analyses evaluated associations between DOBs and ulcer risk.

**Results:**

The optimal pediatric-specific cutoff was 5.285% [Sensitivity 84%, Specificity 90%, area under the curve (AUC) 0.879]. Children with ulcers had significantly higher median DOBs than those without (3.1% vs. 1.9%; *P* < 0.001). A clear dose–response trend was observed across DOB quartiles (*P* < 0.001). Ulcer risk increased with DOB up to approximately 36.39‰, beyond which the risk plateaued.

**Conclusions:**

A DOB cutoff of 5.285‰ provides excellent diagnostic accuracy for pediatric *H. pylori* infection. Higher DOBs correlate strongly with increased bacterial load, mucosal inflammation, and peptic ulcer (PU) risk up to ∼36.39‰, indicating a saturation effect. Quantitative DOB thus offers diagnostic and prognostic utility, supporting its integration into regional pediatric guidelines.

## 1 Background

*Helicobacter pylori* (H. pylori) infection, the most prevalent chronic bacterial infection worldwide, is typically acquired during early childhood, with a notable increase in prevalence after infancy, particularly after one year of age (1). Prevalence in children ranges from 5% in high-income settings to 40%–50% in low- and middle-income countries, and can exceed 50% in areas with a high incidence of gastric cancer such as central-southern China (2). Intra-familial transmission—especially from mothers or siblings—is the dominant route (3). While most infected children remain asymptomatic, chronic infection predisposes them to gastritis, peptic ulcer(PU)s, mucosa-associated lymphoid tissue lymphoma, and potentially gastric carcinoma in adulthood (4, 5).

To date, no systematic epidemiological investigation has been conducted regarding pediatric H. pylori infection in Fuzhou, a coastal city situated within the high-risk gastric cancer belt of southeastern China (6). The scarcity of data is exacerbated by limited acceptance of invasive diagnostic procedures such as endoscopy in pediatric populations; consequently, early H. pylori infection—an essential determinant for future malignancy risk—is frequently undiagnosed or under-reported (7). Defining local epidemiology is therefore essential for targeted prevention.

The ¹³C-UBT relies on the detection of ¹³CO₂ in exhaled breath after ingestion of ¹³C-urea. The resultant delta over baseline (DOB) value quantifies urease activity and, indirectly, bacterial load (8). The test is painless, radiation-free, and particularly attractive for children (9, 10). However, diagnostic cut-off values (commonly 3.5%–4.0%) established from adult populations are often applied to children without pediatric-specific validation, potentially affecting diagnostic accuracy and clinical decisions (11). Adult data suggest that higher DOB values correlate with greater bacterial density, more severe neutrophilic inflammation (12) and, ultimately, peptic-ulcer risk (7, 13), but comparable evidence in children is limited. A recent Israeli cohort further showed that eradication success was significantly higher when baseline DOB ≥ 70% (75.0% vs. 65.3%, OR = 1.59) (14), implying that DOB may serve not only as a diagnostic but also as a prognostic biomarker. Nevertheless, whether similar correlations between DOB values, bacterial load, inflammation severity, and ulcer risk are applicable to Chinese pediatric populations has yet to be elucidated, highlighting the need for targeted local research (15).

## 2 Material and methods

### 2.1 Study design

This retrospective study enrolled consecutive pediatric patients who underwent the ¹³C-urea breath test (¹³C-UBT), upper gastrointestinal endoscopy, and gastric mucosal histopathology between May 2021 and May 2025 at Fujian Children's Hospital. Breath tests were conducted by dedicated nurses. After fasting for at least 12 h, patients ingested 75 mg of ¹³C-labeled urea dissolved in 80–100 ml water as a test meal, consistent with the latest pediatric UBT guidelines (7). Samples were analyzed by infrared spectroscopy using the **HY-IREXBplus infrared analyzer** (*Huayou Mingkang Optoelectronics Co., Ltd., Guangzhou, China*) together with the **¹³C-urea breath test kit (50 mg)** (*Youlixian, Beijing HuanGeng AnBang Biotechnology Co., Ltd., Beijing, China*). Tests were performed according to the manufacturer's protocol after ≥12 h fasting. The ratio of expired ^13^C and ^12^C measured in parts per thousand was obtained at baseline and 30 min following ingestion of ^13^C-urea (T30-T0). The final result was expressed as the difference between the two scores, delta over baseline (DOB). A cutoff 4.0 DOB was used in accordance with the manufacturer's specifications (16, 17). An increase above 4.0 DOB was considered positive for the presence of H. pylori infection.

The study was approved by the Ethics Committee of Fujian Children's Hospital. The study protocol was approved by the Institutional Ethics Committee of Fujian Children's Hospital (Approval No. 2025ETKLRK06015). All patients provided written informed consent signed by their guardians. We confirm that all methods were performed in accordance with the ethical standards laid down in the Declaration of Helsinki and its later amendments or similar ethical standards.

### 2.2 Study participants

**Figure 1:**
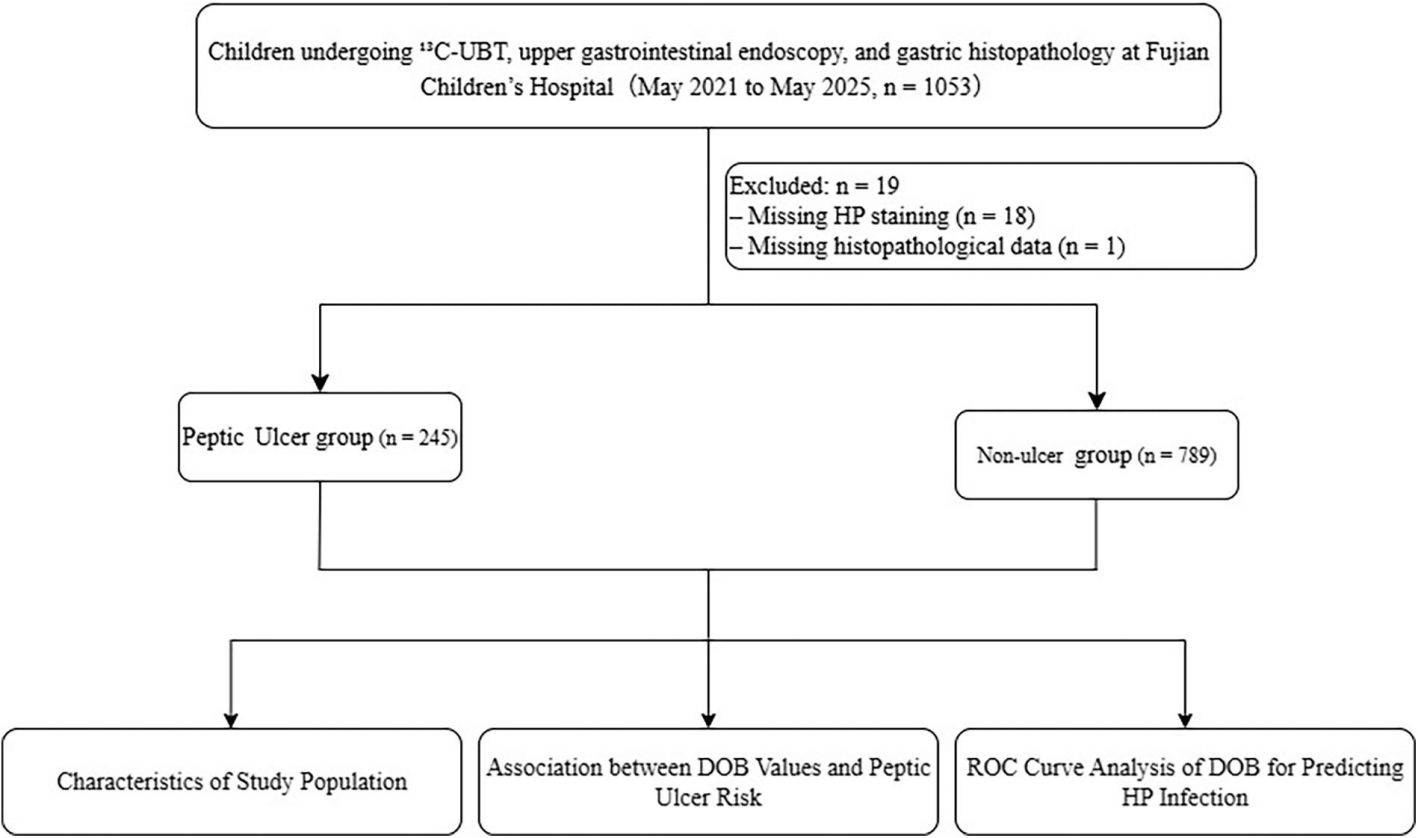
Flowchart of the present study. A total of 1,034 patients were included in this study after the exclusion of 19 individuals. Based on the final clinical diagnosis, the patients were categorized into the Ulcer and Non-ulcer groups, and all subsequent analyses were conducted accordingly.

We retrospectively reviewed consecutive children who underwent ^13^C-UBT, upper endoscopy, and gastric mucosal histopathology at the Gastrointestinal Endoscopy Center of Fujian Children's Hospital from May 2021 to May 2025. 1,034 children met the 2022 Chinese consensus inclusion and exclusion criteria and were included in the final analysis (Figure 1) (18). Inclusion: (1) age 3–18 years; (2) 13C-UBT, endoscopy, and histopathology performed within one month of each other. Exclusion: (1) PPI or H2-receptor antagonist use within 2 weeks; (2) antibiotics within 1 month; (3) prior H. pylori eradication; (4) gastric surgery, gastric malignancy, or concomitant conditions (cirrhosis, uremia, NSAID use) that could alter gastric pathology.

### 2.3 Data collection

1. Clinical data: sex, age, endoscopy report, and mucosal histopathology.
2. H. pylori infection: According to the 2022 Chinese consensus on pediatric (18), diagnosis can be established when any one of the following criteria is fulfilled: (i) positive H. pylori culture; (ii) concordant positive histology and rapid urease test (RUT); (iii) discordant histology/RUT followed by a positive non-invasive test such as the ¹³C-urea breath test (UBT) or stool antigen; or (iv) positive histology or RUT in the context of bleeding peptic ulcer.
3. Endoscopic classification: lesions were graded according to the 2004 Chinese Society of Digestive Endoscopy criteria (19). When ≥2 patterns coexisted, the dominant finding determined classification. Types included superficial (erythematous), hemorrhagic, and erosive gastritis, which were collectively labelled non-atrophic; atrophic gastritis was recorded separately.
4. Histopathology: Biopsies were systematically obtained from gastric antrum and corpus according to the Updated Sydney system, and evaluated based on the latest 2022 Chinese guidelines for histopathological assessment of chronic gastritis (20). Inflammation, atrophy, and intestinal metaplasia were graded on a 0–+++ scale: 0 (absent),+(mild), ++ (moderate), +++ (severe). H. pylori density was assessed by methylene blue staining and graded as 0 (none), +, ++, or +++ according to the Updated Sydney system.

### 2.4 Covariates

Age and gender were included as potential covariates affecting H. pylori infection.

### 2.5 Statistical analysis

Continuous variables were assessed for normality using the Shapiro–Wilk test and presented as mean ± SD or median (IQR), as appropriate. Differences among groups were analyzed by Student t test or one-way Analysis of Variance (ANOVA). Qualitative data were described as *n* (percentages, %) and analyzed using Chi-square test or Fisher's exact test as indicated.

Analyses were conducted using R 4.2.2 (http://www.R-project.org, The R Foundation) and Free Statistics software versions 1.8(FreeClinical, Beijing, China). The *P*-value reported was two-sided, and a value of less than 0.05 was considered statistically significant.

## 3 Results

### 3.1 Baseline characteristics

The demographic and clinical characteristics of the study population are summarized in Table 1. A total of 1,034 children were included, among whom 245 (23.7%) were diagnosed with peptic ulcer. When stratified into three age groups (≤6, 6–12, and >12 years), most participants were school-aged (50.8%), followed by adolescents (46.3%) and preschool children (2.9%). The prevalence of ulcer did not differ significantly among these age groups (*P* = 0.146). However, age distribution differed significantly between H. pylori-positive and negative children, with a higher proportion of adolescents in the Hp (±) group (52.4% vs. 44.1%) and a lower proportion of preschool children (0.7% vs. 3.7%, *P* = 0.006, Supplementary Table S2).

**Table 1:** Demographic and clinical characteristics of patients according to peptic ulcer Status.

Variables	Total (*n* = 1,034)	Non-ulcer group (*n* = 789)	Ulcer group (*n* = 245)	*P*
Age, *n* (%)				0.146
≤6	30 (2.9)	22 (2.8)	8 (3.3)	
6–12	525 (50.8)	414 (52.5)	111 (45.3)	
>12	479 (46.3)	353 (44.7)	126 (51.4)	
Sex, *n* (%)				**<0.001**
Girls	461 (44.6)	397 (50.3)	64 (26.1)	
Boys	573 (55.4)	392 (49.7)	181 (73.9)	
DOB, Median (IQR)	2.0 (0.5, 15.3)	1.9 (0.4, 5.4)	3.1 (0.8, 24.3)	**<0.001**
UBT result, *n* (%)				**<0.001**
Negative	694 (67.1)	558 (70.7)	136 (55.5)	
Positive	340 (32.9)	231 (29.3)	109 (44.5)	
H. pylori staining, *n* (%)				**<0.001**
Negative	759 (73.4)	611 (77.4)	148 (60.4)	
Positive	275 (26.6)	178 (22.6)	97 (39.6)	
Chronic inflammation, *n* (%)				**<0.001**
Mild	906 (87.6)	714 (90.5)	192 (78.4)	
Moderate-Severe	128 (12.4)	75 (9.5)	53 (21.6)	
Acute inflammation, *n* (%)				**<0.001**
None	873 (84.4)	696 (88.2)	177 (72.2)	
Mild	136 (13.2)	83 (10.5)	53 (21.6)	
Moderate-Severe	25 (2.4)	10 (1.3)	15 (6.1)	

Data presented as *n* (%) or median (interquartile range). DOB, delta over baseline; UBT, urea breath test. Comparisons performed using Chi-square or Fisher's exact tests.

Median DOB values were significantly higher in patients with ulcers than those without [3.1‰ [IQR: 0.8–24.3] vs. 1.9‰ [IQR: 0.4–5.4], *P* < 0.001]. Additionally, ulcer patients had significantly higher proportions of H. pylori-positive staining (39.6% vs. 22.6%), positive urea breath tests (44.5% vs. 29.3%), moderate-to-severe chronic inflammation (21.6% vs. 9.5%), and acute inflammation (27.7% vs. 11.8%, all *P* < 0.001). Boys accounted for significantly more ulcer cases than girls (73.9% vs. 26.1%, *P* < 0.001).

When stratified by DOB quartiles, a clear dose-response trend emerged, showing increasing prevalence of ulcers, H. pylori positivity, and moderate-to-severe inflammation scores with higher DOB quartiles (all *P* < 0.001 for trend, Supplementary Table S1 and Supplementary Figures S1, S2). Age distribution across DOB quartiles is also presented in Supplementary Table S1. H. pylori-positive children exhibited substantially higher median DOB values compared to negative children [27.2% [IQR: 14.3–47.2] vs. 1.4% [IQR: 0.3–2.6], *P* < 0.001], and correspondingly greater rates of peptic ulcers and inflammation severity (all *P* < 0.001, Supplementary Table S2).

### 3.2 Association between DOB and peptic ulcer risk

**Figure 2:**
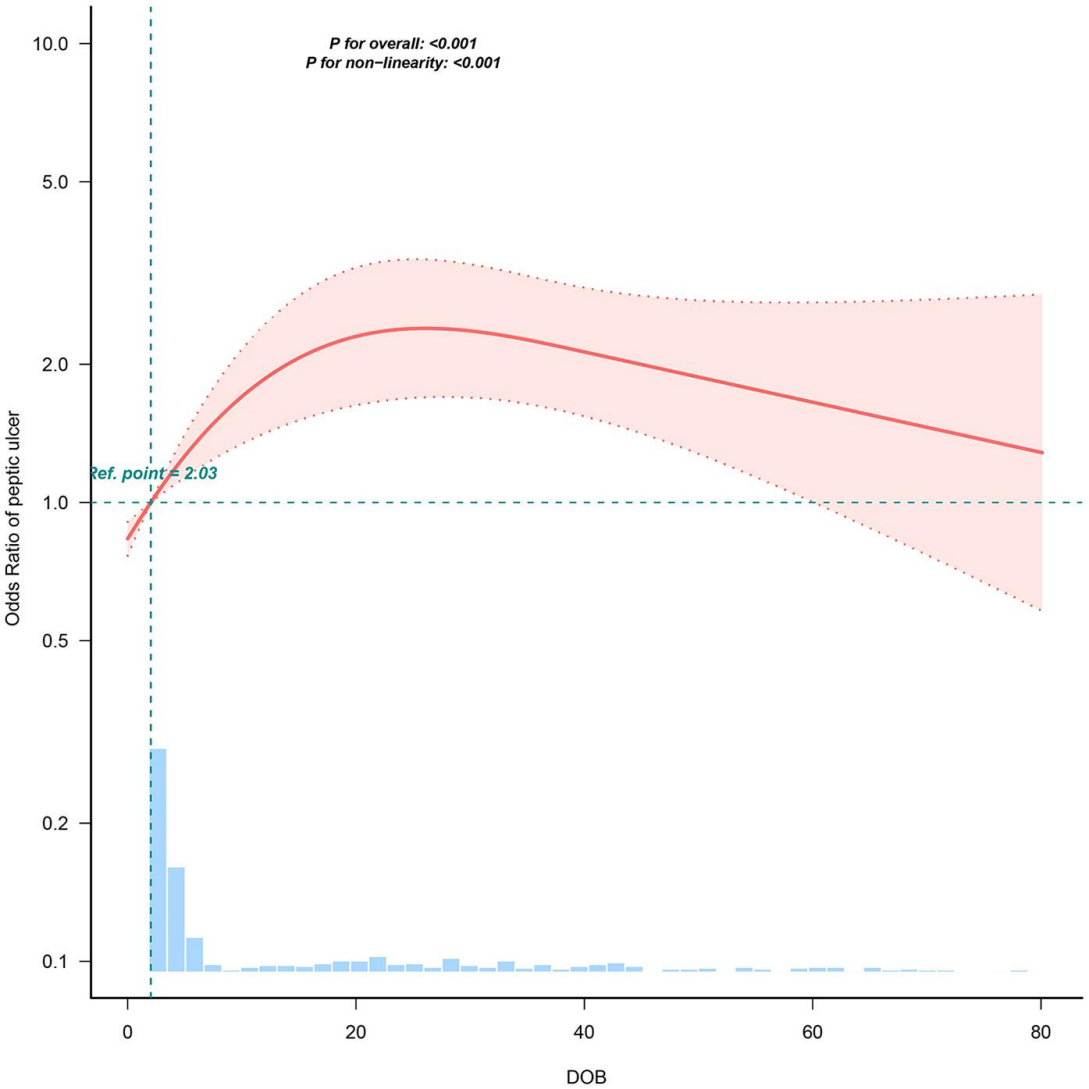
Non-linear relationship between DOB values and peptic ulcer risk. Solid line indicates odds ratio adjusted for age and sex, with shaded area representing 95% confidence interval.

Multivariate logistic regression analysis indicated DOB was independently associated with increased peptic ulcer risk (adjusted OR: 1.01, 95% CI: 1.00–1.02, *P* = 0.003) (Table 2). There was a clear dose-response relationship between DOB and ulcer risk; specifically, patients in the highest DOB quartile (Q4: ≥15.34%) exhibited significantly higher ulcer risk compared with those in the lowest quartile (Q1: 0%–0.46%; adjusted OR: 2.20, 95% CI: 1.45–3.33, *P* < 0.001). Accordingly, the association between DOB and peptic ulcer risk showed a non-linear curve in RCS analysis (*P* < 0.001, Figure 2). Threshold analysis identified an inflection point at a DOB value of 36.39% (Table 3), below which DOB significantly increased ulcer risk (adjusted OR: 1.038, 95% CI: 1.022–1.055, *P* < 0.001), while above this threshold, no further increase in risk was observed (adjusted OR: 0.99, 95% CI: 0.955–1.026, *P* = 0.585).

**Table 2:** Association between DOB and peptic ulcer risk.

Variable	No. of peptic ulcer, %	Crude		Adjusted^a^	
		OR (95%CI)	*P*	OR (95%CI)	*P*
DOB (%)	245 (23.7)	1.01 (1–1.02)	**0**.**003**	1.01 (1–1.02)	**0**.**003**
Q1 (0–0.46)	48 (18.6)	1 (Ref)		1 (Ref)	
Q2 (0.46–2.045)	50 (19.3)	1.05 (0.67–1.63)	0.839	1.07 (0.68–1.67)	0.78
Q3 (2.045–15.34)	60 (23.3)	1.33 (0.87–2.03)	0.195	1.33 (0.86–2.06)	0.198
Q4 (15.34–89.75)	87 (33.6)	2.21 (1.47–3.32)	**<0**.**001**	2.2 (1.45–3.33)	**<0**.**001**
Trend. test	245 (23.7)	1.32 (1.15–1.5)	<0.001	1.31 (1.15–1.5)	<0.001

n in this table indicates the number of children. ^a^Model adjusted age, sex.

**Table 3:** Threshold effect analysis of the relationship of DOB with peptic ulcer.

Inflection point	Adjusted OR (95% CI)	*P*-value
<36.39	1.038 (1.022–1.055)	**<0** **.** **001**
≥36.39	0.99 (0.955–1.026)	0.5851
Likelihood ratio test		**0**.**018**

OR, odds ratio; CI, confidence interval. Adjusted for age and sex.

### 3.3 ROC curve analysis of DOB for predicting *H. pylori* Infection

**Figure 3:**
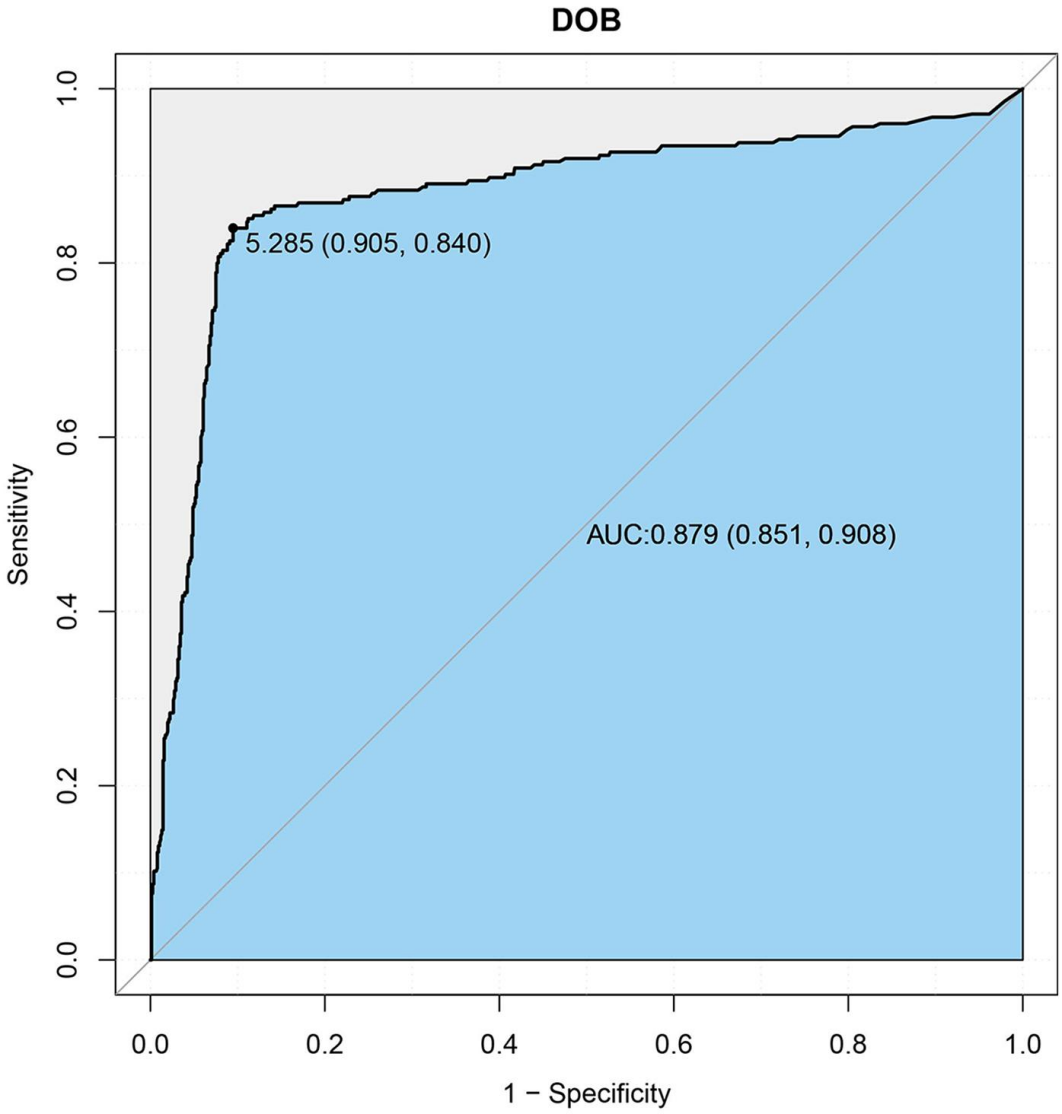
Receiver operating characteristic (ROC) curve of DOB for predicting H. pylori Infection. Area Under the Curve (AUC) = 0.879; Optimal DOB cut-off = 5.285%.

ROC curve analysis demonstrated that DOB values exhibited excellent diagnostic accuracy for predicting H. pylori infection, yielding an area under the curve (AUC) of 0.879 (95% CI: 0.851–0.908), according to accepted diagnostic accuracy criteria (21). The optimal pediatric-specific cutoff was 5.285%, with a sensitivity of 84.0% and specificity of 90.5% (Figure 3).

## 4 Discussion

Our study addresses a critical knowledge gap by establishing a pediatric-specific ¹³C-UBT cut-off value of 5.285% for H. pylori detection in Fuzhou, achieving high sensitivity (84%) and specificity (90%). This threshold is higher than the adult standard (3.5–4.0%), but comparable to the pediatric-specific values (5.0–5.5%) identified previously in cohorts from Shanghai and Hong Kong (22–24), highlighting the necessity for regional validation due to physiological differences such as faster gastric emptying and lower endogenous CO₂ production in children (25). In the remainder of the Discussion, this value will be referred to as “the pediatric-specific threshold” to avoid repetition.

We further identified a clear dose–response relationship between DOB quartiles and both histological severity and peptic ulcer prevalence, consistent with adult observations by Herold and Becker et al. (24). Notably, peptic ulcer risk plateaued at DOB values above 36.39%, suggesting a “saturation threshold” where further bacterial load increments no longer intensify mucosal injury. This non-linear association potentially explains conflicting results from adult studies that used DOB as a bacterial load marker (26, 27).

Our findings confirm the established consensus that pediatric H. pylori infection significantly elevates peptic ulcer risk, particularly duodenal ulcers (18, 28). Remarkably, the prevalence of peptic ulcers in our Fuzhou pediatric cohort (35.3%) markedly exceeds the 10.6% reported in European multicenter studies of H. pylori-positive children, which were also predominantly duodenal ulcers (8, 29).

Additionally, consistent with prior studies (30), we observed a notable male predominance, suggesting demographic, genetic, or environmental factors influencing susceptibility. When stratified by age groups (<6, 6–12, and >12 years), no statistically significant differences in ulcer prevalence were observed (*P* = 0.146). The feasibility of the ¹³C-UBT in preschool children (<6 years) is limited by compliance and cooperation, which may reduce test accuracy and explain lower DOB values observed in some ulcer cases. In our cohort, preschoolers represented only 2.9% of the population, limiting statistical power; nevertheless, reporting stratified results provides context and highlights the need for further age-specific validation.

This stepwise increase in ulcer prevalence, concurrent with elevations in DOB, bacterial density, and inflammation severity, supports the concept that increased bacterial load exacerbates acute mucosal damage and may elevate long-term malignancy risk (31).

Our findings hold direct implications for clinical practice. Firstly, implementing a pediatric-specific DOB threshold of 5.285‰ can substantially reduce false-negative diagnoses, enhancing clinical management without compromising specificity (7). Secondly, clinicians should consider children with DOB values ≥15% (highest quartile) as high-risk for ulcers, warranting prompt endoscopic evaluation even in minimally symptomatic cases. Thirdly, higher DOB values have been associated with improved eradication outcomes in prior studies (14, 32). While this association may suggest a link between bacterial load and treatment response, our study did not evaluate therapeutic outcomes. Therefore, these observations should be interpreted only as hypothesis-generating and require prospective validation before informing treatment decisions.

Regional variations in DOB thresholds likely reflect differences in epidemiology, H. pylori strain virulence, dietary patterns, socioeconomic status, and genetic predispositions (15, 30). Fuzhou, situated within China's high-risk gastric cancer belt, shares dietary and genetic characteristics with other southeastern Chinese populations, but significantly differs from Western or other Asian populations regarding infection prevalence and clinical outcomes (33). Such disparities underline the importance of region-specific pediatric DOB thresholds rather than universally applying adult or foreign standards.

Mechanistically, elevated DOB values reflect heightened urease activity indicative of increased bacterial load and mucosal inflammation. Previous studies highlight virulence factors such as cagA and vacA genotypes significantly influencing bacterial density and host inflammatory responses (34). Consequently, elevated DOB not only represents bacterial abundance but potentially also pathogen virulence, driving mucosal damage and ulcer formation. The observed plateau at DOB > 36.39% might indicate a saturation threshold of mucosal immune response or bacterial urease activity, beyond which further increases in bacterial load yield minimal incremental tissue injury.

Clinically, recognizing children with elevated DOB values (≥15%) as high-risk could improve timely endoscopic evaluations. Previous studies have suggested a possible relationship between higher DOB values and eradication success rates (7, 32). However, our study did not include treatment outcome data, and thus these findings cannot be extrapolated to guide therapeutic strategies. Future prospective trials are warranted to clarify whether quantitative DOB can serve as a predictor of treatment response.

Future studies should focus on prospective longitudinal cohorts and randomized controlled trials to validate the identified DOB thresholds across diverse pediatric populations. Additionally, integrating molecular characterization of H. pylori strains with quantitative DOB measurements may enhance personalized risk stratification, elucidate pathogen-host interactions, and refine clinical management strategies (7, 32). Ultimately, such comprehensive research could significantly advance pediatric gastroenterology practice.

## 5 Conclusion

In the first endoscopy-confirmed cohort of Fuzhou children, the pediatric-specific threshold of 5.285% optimally detects H. pylori infection. DOB values positively correlate with bacterial load, mucosal inflammation, and ulcer risk up to approximately 36.39%, above which risk plateaus, indicating a saturation effect. These findings support region-specific pediatric guidelines and suggest that quantitative DOB serves as a diagnostic tool and a marker for risk stratification. While associations with bacterial load and ulcer risk are clear, further studies incorporating therapeutic outcomes are needed before drawing conclusions regarding treatment strategies.

## Data Availability

The original contributions presented in the study are included in the article/Supplementary Material, further inquiries can be directed to the corresponding author.
