# Supplementary material for: Quantitative ¹³C-urea breath test values predict peptic ulcer risk in Helicobacter pylori -infected children: a retrospective study
Source: Front Pediatr. 2025 Sep 24;13:1684120. doi: 10.3389/fped.2025.1684120 (PMC12504025; doi:10.3389/fped.2025.1684120)
Supplement: Supplementary file 1 [file Datasheet1.doc]

**Supplementary Figure 1**. Comparison of DOB values among different groups. (A) Peptic ulcer vs. non-ulcer patients; (B) H. pylori-positive vs. negative patients; (C–E) Severity trends of chronic inflammation, acute inflammation, and H. pylori density across DOB quartiles.

**Supplementary Figure 2**.Dose-response Relationship Between DOB and Histopathological Severity.

**Supplementary Table 1**. Demographic and Clinical Characteristics Stratified by DOB Quartiles.

**Supplementary Table 2**. Patient Characteristics Stratified by Helicobacter pylori Infection Status

**
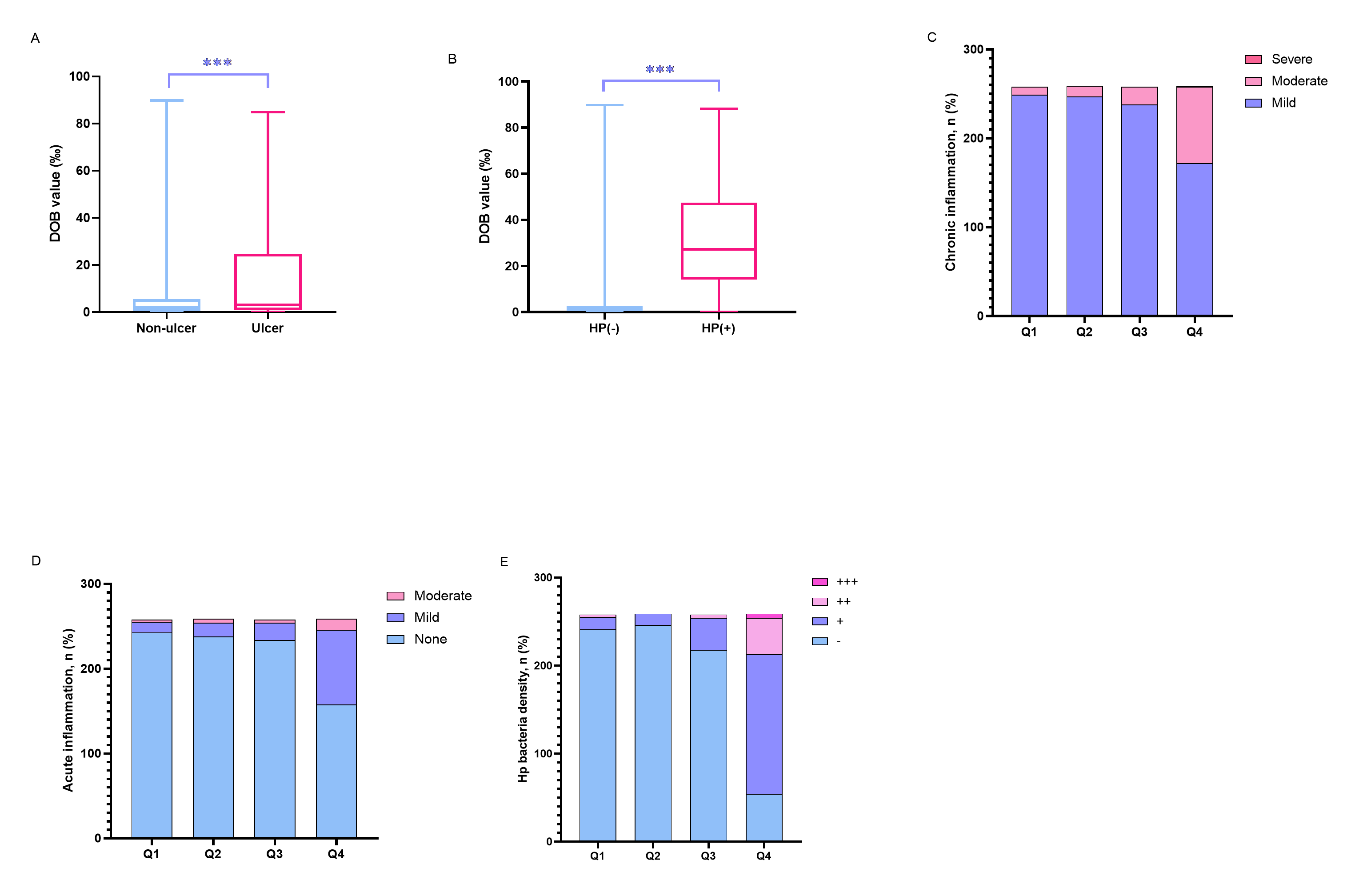
**

**Supplementary Figure 1.** Comparison of DOB values among different groups. (A) Peptic ulcer vs. non-ulcer patients; (B) H. pylori-positive vs. negative patients; (C–E) Severity trends of chronic inflammation, acute inflammation, and H. pylori density across DOB quartiles.

**
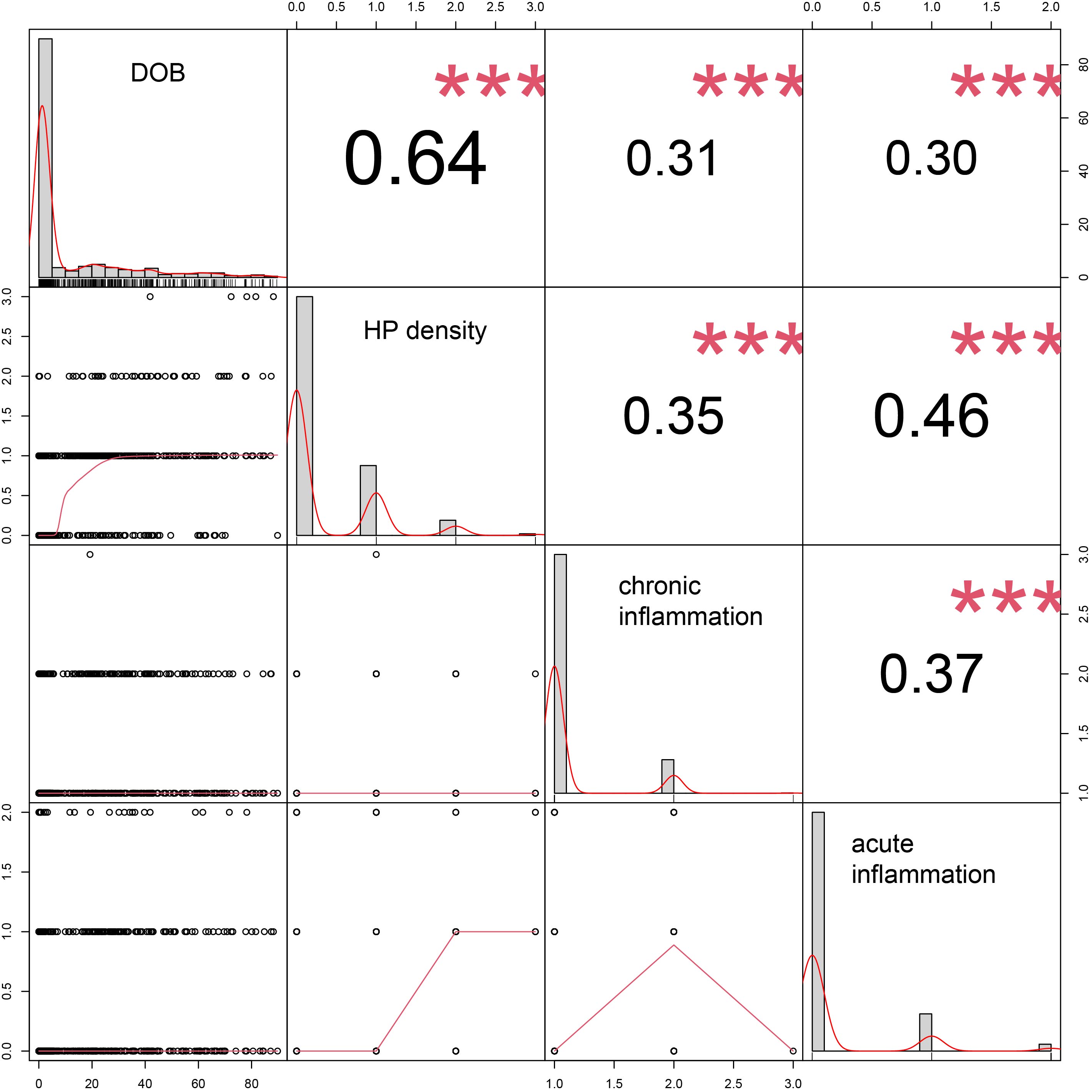
**

**Supplementary Figure 2.** Dose-response Relationship Between DOB and Histopathological Severity.

Supplementary Table 1. Demographic and Clinical Characteristics Stratified by DOB Quartiles.

| Variables | DOB, ‰ | | | | | |
| --- | --- | --- | --- | --- | --- | --- |
|  | Total | **Q1**  (0-0.46) | **Q2** (0.46-2.045) | **Q3** (2.045-15.34) | **Q4** (15.34-89.75) | *P* |
| NO. | 1034 | 258 | 259 | 258 | 259 |  |
| Age, n (%) |  |  |  |  |  | **0.02** |
| ≤6 | 30 ( 2.9) | 4 (1.6) | 14 (5.4) | 10 (3.9) | 2 (0.8) |  |
| 6-12 | 525 (50.8) | 138 (53.5) | 126 (48.6) | 136 (52.7) | 125 (48.3) |  |
| >12 | 479 (46.3) | 116 (45) | 119 (45.9) | 112 (43.4) | 132 (51) |  |
| Sex, n (%) |  |  |  |  |  | 0.751 |
| Girls | 461 (44.6) | 117 (45.3) | 121 (46.7) | 114 (44.2) | 109 (42.1) |  |
| Boys | 573 (55.4) | 141 (54.7) | 138 (53.3) | 144 (55.8) | 150 (57.9) |  |
| 1. pylori staining,   n (%) |  |  |  |  |  | **< 0.001** |
| Negative | 759 (73.4) | 241 (93.4) | 246 (95) | 218 (84.5) | 54 (20.8) |  |
| Positive | 275 (26.6) | 17 (6.6) | 13 (5) | 40 (15.5) | 205 (79.2) |  |
| Ulcer, n (%) |  |  |  |  |  | **< 0.001** |
| No | 789 (76.3) | 210 (81.4) | 209 (80.7) | 198 (76.7) | 172 (66.4) |  |
| Yes | 245 (23.7) | 48 (18.6) | 50 (19.3) | 60 (23.3) | 87 (33.6) |  |
| Ulcer site, n (%) |  |  |  |  |  | **< 0.001** |
| None | 789 (76.3) | 210 (81.4) | 209 (80.7) | 198 (76.7) | 172 (66.4) |  |
| GU | 8 ( 0.8) | 3 (1.2) | 1 (0.4) | 4 (1.6) | 0 (0) |  |
| DU | 224 (21.7) | 39 (15.1) | 47 (18.1) | 55 (21.3) | 83 (32) |  |
| Combined ulcer | 13 ( 1.3) | 6 (2.3) | 2 (0.8) | 1 (0.4) | 4 (1.5) |  |
| Hp bacteria density, n (%) |  |  |  |  |  | **< 0.001** |
| - | 759 (73.4) | 241 (93.4) | 246 (95) | 218 (84.5) | 54 (20.8) |  |
| + | 222 (21.5) | 14 (5.4) | 13 (5) | 36 (14) | 159 (61.4) |  |
| ++ | 48 ( 4.6) | 3 (1.2) | 0 (0) | 4 (1.6) | 41 (15.8) |  |
| +++ | 5 ( 0.5) | 0 (0) | 0 (0) | 0 (0) | 5 (1.9) |  |
| Chronic inflammation, n (%) |  |  |  |  |  | **< 0.001** |
| + | 906 (87.6) | 249 (96.5) | 247 (95.4) | 238 (92.2) | 172 (66.4) |  |
| ++ - +++ | 128 (12.4) | 9 (3.5) | 12 (4.6) | 20 (7.8) | 87 (33.6) |  |
| Acute inflammation, n (%) |  |  |  |  |  | **< 0.001** |
| - | 873 (84.4) | 243 (94.2) | 238 (91.9) | 234 (90.7) | 158 (61) |  |
| + | 136 (13.2) | 12 (4.7) | 16 (6.2) | 20 (7.8) | 88 (34) |  |
| ++ - +++ | 25 ( 2.4) | 3 (1.2) | 5 (1.9) | 4 (1.6) | 13 (5) |  |

DOB quartiles defined as Q1 (0–0.46‰), Q2 (0.46–2.045‰), Q3 (2.045–15.34‰), Q4 (15.34–89.75‰).Comparisons were made using Chi-square or Fisher’s exact test. GU, Gastric Ulcer; DU, Duodenal Ulcer; "+", positive grading of inflammation or bacterial density.

Supplementary Table 2. Patient Characteristics Stratified by *Helicobacter pylori* Infection Status

| Variables | Total (n = 1034) | Hp(-)  (n = 759) | Hp(+)  (n = 275) | *p* |
| --- | --- | --- | --- | --- |
| Age, n (%) |  |  |  | 0.006 |
| ≤6 | 30 ( 2.9) | 28 (3.7) | 2 (0.7) |  |
| 6-12 | 525 (50.8) | 396 (52.2) | 129 (46.9) |  |
| >12 | 479 (46.3) | 335 (44.1) | 144 (52.4) |  |
| Sex, n (%) |  |  |  | 0.932 |
| Girls | 461 (44.6) | 339 (44.7) | 122 (44.4) |  |
| Boys | 573 (55.4) | 420 (55.3) | 153 (55.6) |  |
| DOB, Median (IQR) | 2.0 (0.5, 15.3) | 1.4 (0.3, 2.6) | 27.2 (14.3, 47.2) | < 0.001 |
| UBT result, n (%) |  |  |  | < 0.001 |
| Negative | 694 (67.1) | 655 (86.3) | 39 (14.2) |  |
| Positive | 340 (32.9) | 104 (13.7) | 236 (85.8) |  |
| Ulcer, n (%) |  |  |  | < 0.001 |
| No | 789 (76.3) | 611 (80.5) | 178 (64.7) |  |
| Yes | 245 (23.7) | 148 (19.5) | 97 (35.3) |  |
| Ulcer site, n (%) |  |  |  | < 0.001 |
| None | 789 (76.3) | 611 (80.5) | 178 (64.7) |  |
| GU | 8 ( 0.8) | 8 (1.1) | 0 (0) |  |
| DU | 224 (21.7) | 133 (17.5) | 91 (33.1) |  |
| Combined ulcer | 13 ( 1.3) | 7 (0.9) | 6 (2.2) |  |
| Chronic inflammation, n (%) |  |  |  | < 0.001 |
| + | 906 (87.6) | 729 (96) | 177 (64.4) |  |
| ++-+++ | 128 (12.4) | 30 (4) | 98 (35.6) |  |
| Acute inflammation, n (%) |  |  |  | < 0.001 |
| - | 873 (84.4) | 720 (94.9) | 153 (55.6) |  |
| + | 136 (13.2) | 29 (3.8) | 107 (38.9) |  |
| ++-+++ | 25 ( 2.4) | 10 (1.3) | 15 (5.5) |  |

GU, Gastric Ulcer; DU, Duodenal Ulcer; "+", positive grading of inflammation.
